# Supplementary material for: Chromothripsis during telomere crisis is independent of NHEJ, and consistent with a replicative origin
Source: Genome Res. 2019 May;29(5):737–49. doi: 10.1101/gr.240705.118 (PMC6499312; doi:10.1101/gr.240705.118)
Supplement: Supplemental Material [file supp_gr.240705.118_Supplemental_file_1.zip › contigs/annotated_contigs/DB57/contig.2.DB57_length_294_mean_cov_2.55102040816.docx]

**DB57_length_294_mean_cov_2.55102040816**

```
AGCTTATGGAAGTGAAAAAAACTCGGGTTTTAAAGCCACCTACACACTGTATACTCTTAGACAAGTTACTCAATGTCGCCAAGCTTTGT
 >chr1:174552994-174553154 - E=4e-85
TTTCTTCATTTCCCCTACTAACCCACCACAATTGTGATGCTAATTCTAATTTTCAAAATTGTTTTAA|GATT|TTTTGTATCAATGTTC
                                                                    >chr1:174566270-174
GTCAGAAATGTTGGCCTGGAATTTTCTTTTTTTGATGTGTCTTTGTCTGGTTTTGTTATCAGGGTAATACTGCCCTCATAGAATGAGTT
566408 + E=8e-72
TGGAAGTATTCACTCCTCCATTATTTTTT
```
